# Aligning the Economic Value of Companion Diagnostics and Stratified Medicines 

**DOI:** 10.3390/jpm2040257

**Published:** 2012-11-26

**Authors:** Edward D. Blair, Elyse K. Stratton, Martina Kaufmann

**Affiliations:** Integrated Medicines Ltd., Topfield House, Ermine Street, Caxton, Cambridge, CB23 3PQ, UK; E-Mails: elyse.stratton@integratedmedicines.co.uk (E.K.S.); martina.kaufmann@integratedmedicines.co.uk (M.K.)

**Keywords:** stratified medicines, economic value, health outcomes, multiple stakeholders, companion diagnostic, regulation, reimbursement

## Abstract

The twin forces of payors seeking fair pricing and the rising costs of developing new medicines has driven a closer relationship between pharmaceutical companies and diagnostics companies, because stratified medicines, guided by companion diagnostics, offer better commercial, as well as clinical, outcomes. Stratified medicines have created clinical success and provided rapid product approvals, particularly in oncology, and indeed have changed the dynamic between drug and diagnostic developers. The commercial payback for such partnerships offered by stratified medicines has been less well articulated, but this has shifted as the benefits in risk management, pricing and value creation for all stakeholders become clearer. In this larger healthcare setting, stratified medicine provides both physicians and patients with greater insight on the disease and provides rationale for providers to understand cost-effectiveness of treatment. This article considers how the economic value of stratified medicine relationships can be recognized and translated into better outcomes for all healthcare stakeholders.

## 1. Introduction

For many years, pharmaceutical companies have struggled with declining productivity, increasing development costs and attrition of traditional blockbuster medicines. The impact being that as clinical failure rates increased, so did competition from generics and biosimilars. Healthcare authorities were also introducing increased price scrutiny and seeking ways to limit high payments for high-volume products. 

Stratified medicines (those medicines that treat a particular disease form that is described in part by a companion diagnostic test, either by improved efficacy or reduced side-effects) offer one potential solution to these challenges, while also offering considerable benefits in terms of patient outcomes. The use of biomarkers to identify patients in clinical studies has been shown to both reduce the size and duration of clinical studies, with considerable development cost benefits as well as a higher likelihood of success / reduced attrition rate [1]. The transition of biomarkers to validated indicators of clinical outcome, in the form of approved companion diagnostics [any diagnostic test (molecular, immuno, point-of-care, tissue-based, *etc.*) that is used to identify patients who will benefit by improved safety or response to a stratified medicine], can help to achieve premium pricing, improve market uptake, market size and market share in such a way that blockbuster revenues are retained [2,3,4,5]. In addition, there is also evidence that companion diagnostics can protect proprietary medicines from generics late in the product lifecycle [6]. 

Despite a wide disparity in relative markets and pricing, the relative economic contributions of the companion diagnostic test to a drug-diagnostic co-development programme, in terms of the impact to the net present value (NPV) of the associated stratified medicine is substantial [7]. The joint diagnostic and drug product also creates new commercial opportunities for strategic pricing and favorable reviews by cost-effectiveness committees, both at the state and private levels [8,9]. Ultimately, stratified medicine should also secure product reimbursement by payors by meeting the standards of clinical and commercial efficacy. What began as a clinical risk-mitigation strategy ultimately provides a transition from the blockbuster model to a more sustainable set of combination products which offer blockbuster income to industry [2,3], while providing clinical and economic outcomes that benefit other key stakeholders.

## 2. Relationship Economics and Relationship Structure

Several articles and economic models [10,11,12] have assessed the economic uplift of companion diagnostics and perceive three life cycles of product management: (1) diagnostic-drug co-development and co-approval; (2) diagnostic introduction post-approval to revitalize drug product utility; and (3) diagnostics used to segment a patient population for better safety, lower risk and/ or greater efficacy [13]. In all cases, both diagnostic and drug developers have gained financially, but the economic benefits have largely aggregated to the pharma partner, while undertaking a regulatory submission for diagnostics providers significantly impacts their operating costs. 

**Figure 1:**
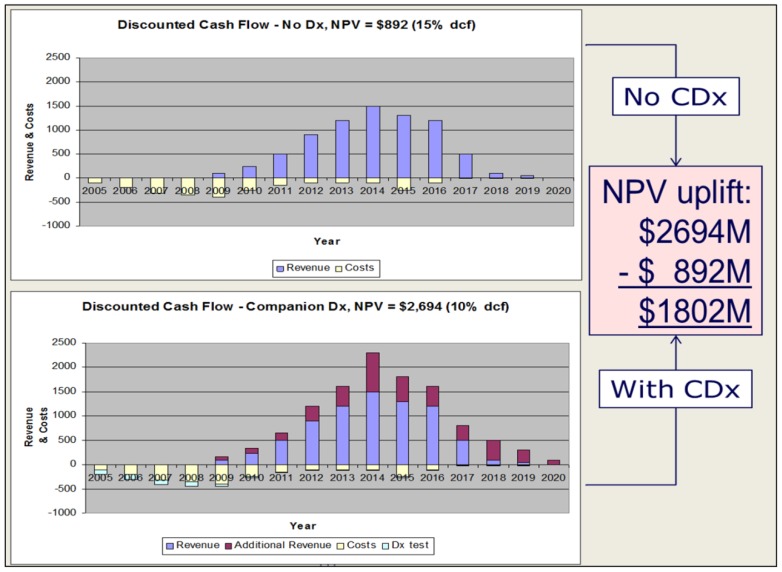
Adapted with permission from Blair E.D. (2008)[7].

While it is recognized that the pharma partner (Rx) and the diagnostic partner (Dx) conduct their business on vastly different scales (Rx $1000bn, relevant Dx $10bn sales per annum [14,15], companion diagnostic deals are very capital-intensive for the diagnostic partner. Economic quantitation of these benefits over a 20-year period suggests a potential net present value (NPV) uplift of $1.8 bn, from $900 M to $2.7 bn [7,16] (see Figure 1). Larger pharma partners recognize that they need to provide financial stability to their test providers to ensure that they are viable through the development and commercialization stages. This has been achieved through secured loans, such as Pfizer’s $25 M investment in Monogram Biosciences [17], or supporting investment across divisions in the case of Roche Pharma and Roche Diagnostics. The availability of internal diagnostics organizations to pharma divisions, such as at Roche, Abbott and Novartis, does not as yet appear to represent a more favourable structure than use of external diagnostics providers, such as at GSK, Pfizer or AstraZeneca.

Companion diagnostic development has therefore been concentrated at a small number of organisations with the appropriate financial stability, technical expertise, regulatory knowledge and global reach for commercialization. Of course, there is no single diagnostic partner that fits all of these requirements, and partnership deals should include resources to supplement the regulatory approval and commercial launch process.

**Figure 2:**
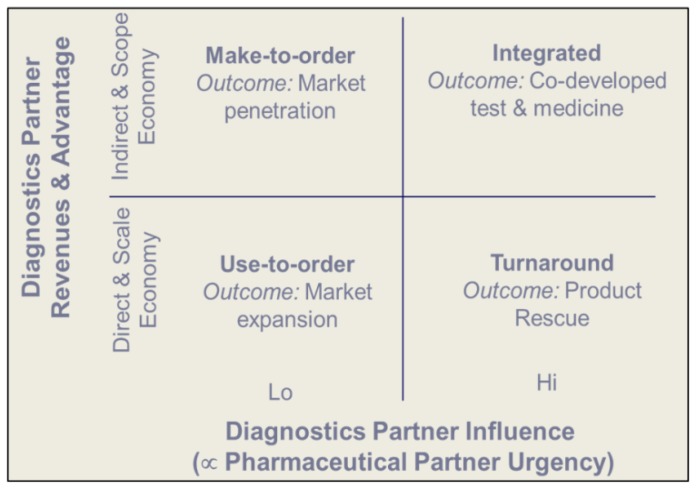
Rx-Dx Model Relationships. Adapted with permission from Blair E.D. (2010)[16].

To more accurately gauge the economic relationship between Rx-Dx partners, we have used value-modeling to look at how simple fee-for-service relationships might translate into risk-sharing relationships [7,10,16]. The structures of these various models are shown in Figure 2. There is a greater risk spread between pharmaceutical probability of success (PoS), estimated at 1-in-60, *vs*. the better than 1-in-5 for the diagnostic, although new regulation may impact on the Dx PoS. Because diagnostics contribute to lowering the clinical and regulatory risk for the pharmaceutical product [5,11,12,13], there could be an economic case for a hybrid fee-for-service relationship with qualified risk sharing reflected through pharma royalty sharing, especially in the case of strong diagnostic IP or a dominant testing market position. This would offset the capital investment by the diagnostic company and could facilitate the commercial success of the co-launch by providing resources to make the test available widely. To date, there are no published reports of such royalty-sharing but this type of relationship has long been debated.

The structure of these relationships should be guided by associating an Rx Product Profile (Target Product Profile, TPP) with a Dx Product Profile (Target Test Profile, TTP) [10], with the latter articulating the technical, clinical and commercial performance of a test that will ensure successful development of a linked stratified medicine described by the TPP.

The economic value of the relationship will also incorporate the contributions of the diagnostic to shorter approval times, premium pricing and clearing reimbursement review hurdles in all payor settings.

## 3. Impact on Regulation, Pricing and Reimbursement

In 2011, multiple product approvals and commercial launches provided clear examples of the benefits of companion diagnostic development and triggered updated regulatory guidance. In the US, the Food and Drug Administration (FDA) issued Draft Guidance on “*In Vitro* Companion Diagnostic Devices” [18] which supported the methodology used in both the Zelboraf [19] and Xalkori [20] regulatory submissions and subsequent companion diagnostic co-approvals in August 2011 [21]. Both products were rewarded with approvals ahead of their PDUFA dates.

In Europe, the European Medicines Agency (EMA) released a White Paper on the use of biomarkers in drug development [22] and the European Commission announced a review and update to the IVD Directive [23]. According to proposed key changes, companion diagnostics are expected to remain under IVD directive and be listed as class C (high individual risk and/or moderate public risk), conforming with the new GHTF (Global Harmonization Task Force) classification model. The implementation of that model would thus bring them under Notified Body review. Whereas public consultation suggested that the proposed demonstration of clinical utility might not be workable for companion diagnostics, closer cooperation between the IVD Medical Device organization and EMA is considered a positive benefit from the IVDD revisions. A draft was due to be released in early 2012, while the timeline for implementation of the revised IVD directive has still to be defined.

Pricing for these targeted therapeutics has been established in context of their narrowly defined patient populations, with the strategic intent to expand markets post-approval. Xalkori (critozinib, Pfizer) is priced at $115,200 per annum (approximately $9,600 per month) [24] and was originally in development as a c-Met inhibitor before the association with the Anaplastic Lymphoma Kinase (ALK) mutation. It is currently in EORTC studies for multiple tumors with MET and/or ALK mutations, as well as separate studies in lymphoma which will expand its “narrow” indication. While the co-approved ALK test, Vysis ALK Break Apart In Situ Hybridisation FISH Probe Kit from Abbott Molecular, is priced higher than similar FISH-based kit tests, at $1500 [25] (the HER2 FISH test is priced closer to $500), the price does not reflect the full value contribution to the successful commercialisation of the associated therapeutic (see Table 1). Approval was based on data of 255 ALK positive patients enrolled in a phase II and a phase I study [26,27,28] meaning that development of Xalkori and its companion diagnostic took only four years from identification of the target to approval [29,30] and with ALK positivity considered to be close to 2%–7% [31], even a highly effective drug would have failed in all-comer trials without patient stratification. In addition to fast approval, this regulatory review paradigm significantly impacts on the size of clinical trial and the associated costs [32,33].

**Table 1:** The Price *vs.* Value Imbalance. * Projected Annual Sales 2012 based on HY12 – roche.com.

Targeted Therapy	Annual Price	Companion Diagnostic	Test Price	Model	Value
Xalkori (critozinib, Pfizer)	$115,200	Vysis ALK Break Apart In Situ Hybridisation FISH Probe Kit (Abbott Molecular)	$1,500	Rescue (ALK positivity ~7%)	TBD
**Zelboraf** (vemurafenib, Plexxikon / Diiachi-Sankyo/ Roche)	$56,400	Cobas 4800 BRAF V600 Mutation Test (Roche Molecular)	$120-$150	Co-development (BRAF V600E mutation ~40%)	$144M ($213M*)
Herceptin (trastuzumab, Genentech / Roche)	$70,000	HercepTest (Dako)	$500	Rescue (HER-2 expression score 3+ ~ 10%)	$620M*

Well ahead of projected view timelines, the FDA also approved Zelboraf (vemurafenib, Plexxikon / Diiachi-Sankyo / Roche) for the treatment of BRAF V600E mutation-positive, inoperable or metastatic melanoma, and co-approved the Cobas 4800 BRAF V600 Mutation Test, a diagnostic test developed by Roche to identify patients eligible for treatment. The pivotal phase III trial was run in a selected patient population of 675 patients with previously untreated, metastatic melanoma with the BRAF V600E mutation and showing a tremendous clinical benefit over chemotherapy [19].

With a prevalence of BRAF mutation in melanoma of about 42% (COSMIC database [34]) and V600E being the most common mutation, the actual clinical benefit would approximately be 50%–60% in an unselected population, so approval might not have been granted. Thus, while identifying the BRAF V600E mutation in metastatic melanoma and pursuing an enrollment strategy based on the Cobas 4800 BRAF V600 Mutation Test by necessity halved the patient population size for vemurafenib early in its development, the strategy also expedited the development timeline and created a new paradigm for phase II/III clinical trial design [19].

Based on the data for progression free survival from the pivotal phase III trials, a 6 months treatment period can be assumed, resulting in costs of Zelboraf treatment in the range of $56,400 ($9.400 per month) [35], depending on the actual treatment duration. Costs for patient selection by the COBAS V600E test are considered to be in the range of $120-$150 [35], but this test price reflects neither the value that the test brings to the medicine, nor the heavily subsidized development costs borne by Roche Diagnostics. Annual sales of Zelboraf are already $200M and so may exceed 2015 estimates of $372 M [36] whereas the test is unlikely to exceed annual sales of $10M by 2015–2019 [37].

## 4. Beyond UK NICE, Health Technology Assessment in Germany

While the higher prices of targeted drugs may conflict with the interests of pricing agencies, there are signs that the agencies may be more amenable to a clearly defined patient population. In its rejection of Yervoy (ipilumumab, Bristol-Myers Squibb)—a human monoclonal antibody for malignant melanoma approved by the FDA in March 2011 [38]—the UK National Institute for Health and Clinical Effectiveness (NICE) calculated that the £80,000 annual cost was too high per QALY gained, but also went on to state, “Unfortunately, no patient characteristics or biomarkers have yet been identified to help identify this small group of people most likely to gain long-term benefit from receiving ipilimumab” [39]. Zelboraf, estimated cost of £37,500 for a course of treatment and with a recognised companion diagnostic, has been under review to determine whether its QALY value merits a positive opinion from NICE; in fact it was recently given a negative opinion by NICE [40]. Given the price of Xalkori (£78,000 per year), the current price of ALK testing (£900) for the ALK testing, and considering the rather low prevalence of ALK translocations, testing all advanced NSCLC patients in order to identify ALK-positive patients might turn out not to be cost effective [41], an issue noted by others [10].

Although regulatory review, pricing and payment committees may all be aware of stratified medicine, there is not yet an integrated system for the process. As an example of the number of agencies involved, we look to Germany’s approach to stratified medicines [42].

Germany, a key pharmaceutical and diagnostic market in Europe with nearly 90% of the population being part of the public insurance system, is struggling to contain healthcare costs and to introduce new technologies clinically- and cost-effectively. 

A dedicated federal committee (Common Health Board, GBA) includes representatives from payers, physicians, hospitals and wider (lay) society, and is taking public system reimbursement decisions based on input from companies, medical societies, and governmental institutions. Such government institutions include an independent organization charged with health technology assessment (HTA) called the Institute for Quality and Efficiency in Healthcare (IQWIG), founded in 2004. Based on requests from GBA, therapies as well as diagnostic tests are evaluated by IQWIG. This process may last 12 months or longer and allows companies to engage with IQWIG at certain step and results in a reimbursement recommendation to GBA. For diagnostic tests the complexity is even higher since final reimbursement may only be approved by the 16 individual German Bundesländer (regions).

Whereas the demonstration of health economic benefit was only introduced to the IQWIG process in 2008, it has now become even more critical for reimbursement of therapeutics due to a recent German health reform [43], based on which the price for therapeutics is determined by a robust cost-benefit analysis. Stratified medicine approaches that provide either enhanced efficacy or improved safety in the respective selected target population may be allowed premium pricing according to this regulation if the cost-benefit analysis is positive.

Companion diagnostic tests on the other hand still lack such a value-based approach. As with other diagnostics, they are covered within the DRG (diagnosis related group) fee schedule in the inpatient (hospital) setting and a code-based fee schedule in the outpatient (ambulatory) setting, respectively, a technical procedural cost which does not consider clinical or economic value.

As Germany is just one of the five major EU markets, the complexities of satisfying EU healthcare stakeholders are complex, evolving and ultimately multiplying. Some headway is being made in this respect through both Europe-wide harmonization of drug and diagnostic regulations, and also many global harmonization activities that include the use of companion diagnostics and stratified medicines [31].

## 5. Conclusions

When personalized medicine strategies were originally developed over a decade ago, there was little commercial enthusiasm for stratifying patients for therapeutic response, but changes in the clinical and commercial environment also make a strong economic case for stratified medicine. Stratified medicines impact the economic relationship of both large pharma and companion diagnostic test developers by establishing economic risk-sharing in test development and trial operations, introducing regulatory strategy for diagnostic developers and building designated markets for final products. The joint diagnostic and drug product also creates new commercial opportunities for strategic pricing and favorable reviews by cost-effectiveness committees, both at the state and private levels. 

Beyond the co-developer relationship, stratified medicines impact healthcare providers at the points of testing, prescribing and payment and deliver more valuable outcomes to patients when they are receiving the appropriate care.

Finally, more timely access to safe, highly-effective patient-stratified therapies moves healthcare towards an era of *predictive* medicine, as opposed to *reactive* medicine, with the undoubted benefits of earlier treatment translating to better long-term outcomes. In this respect, it may well be feasible to prolong life, but the challenge of future healthcare is to ensure that quality of life remains commensurate with this longevity.
